# Modulating reflexes enables speed control in simulated human walking and running

**DOI:** 10.1038/s41598-026-48509-z

**Published:** 2026-04-21

**Authors:** Elsa K. Bunz, Alice J. Bruel, Auke J. Ijspeert, Syn Schmitt

**Affiliations:** 1 Institute for Modelling and Simulation of Biomechanical Systems, University of Stuttgart, 70569 Stuttgart, Germany; 2 Biorobotics Laboratory, Ecole Polytechnique Federale de Lausanne, 1015 Lausanne, Switzerland

**Keywords:** Computational biophysics, Computational models

## Abstract

Human locomotion is characterized by remarkable versatility, enabling smooth speed modulation, acceleration, deceleration, and transitions between gaits. This versatility is thought to emerge from interactions between the musculoskeletal system, spinal circuits—including reflexes and central pattern generators—and supraspinal control. The relative contributions of these components remain a subject of ongoing debate. In this study, we explore the potential role of reflex modulation in achieving the observed flexibility of human locomotion. Using a neuromusculoskeletal model controlled by reflexes between agonist/antagonist muscle groups, we show that a wide range of walking and running speeds can be generated using reflex modulation. We devise a modulation strategy of key reflexes, which allows for setting a desired target speed before simulation (offline modulation) as well as dynamically adjusting speed during run-time (online modulation). Furthermore, we can demonstrate that switching between parameter sets allows for smooth transitions between walking and running. This study aims to understand the capabilities of reflexes as a core component of locomotor control and therefore isolates reflexes from other components of the spinal cord. While the study, thus, does not aim to fully replicate human motor control, it demonstrates that reflex modulation can account for speed control in walking and running as well as gait transitions, highlighting the possible contributions of reflexes to versatile locomotion.

## Introduction

Human locomotion is characterized by different types of gaits and remarkable biomechanical adaptability, enabling individuals to walk and run across a wide range of speeds^1^, with frequent accelerations, decelerations, and transitions between gaits. Each individual has preferred walking and running speeds, influenced by their unique musculoskeletal properties and muscle recruitment strategies^2,3^.

A central question in motor neuroscience is how such versatility is achieved. Locomotion emerges from the complex interaction of the musculoskeletal system, the environment and the neural system—including spinal circuits and supraspinal inputs. Within the mammalian spinal cord, two main mechanisms are thought to contribute to locomotor control: spinal reflexes, which mediate fast responses to proprioceptive signals, and central pattern generators (CPGs), which can generate rhythmic activity in the absence of sensory input but are strongly modulated by sensory feedback in intact mammals^4,5^. Both reflexes and CPGs are further modulated by descending inputs^4,6–8^.

Ijspeert and Daley^9^ hypothesize that the relative contributions of spinal sensing and reflexes, CPGs and descending modulation differ among species, with humans relying more heavily on spinal reflexes and descending modulation rather than on intrinsic rhythm generation. Disentangling these components requires understanding the capabilities of each mechanism in isolation. While CPGs have been isolated experimentally and studied in detail^10,11^, studying the capabilities of reflexes in isolation is not feasible experimentally. It therefore remains an open question to what extent spinal reflexes alone could support the versatility of human locomotion, including speed modulation and gait transitions.

Despite their well-established role in supporting locomotion, the specific contribution of spinal reflexes to speed modulation and gait transitions in humans remains unclear. Experimental studies have shown that spinal reflexes are modulated by higher neural mechanisms allowing for adaptability during different movements, especially during different gait phases^6–8,12,13^.

Although there is no direct evidence that spinal reflex modulation affects gait speed, several works have studied adaptations of spinal reflexes in response to variations in gait speed^14–17^. Prochazka et al.^18^ and Yakovenko et al.^19^ also demonstrated that the contribution of stretch reflexes in load compensation during gait increases with decreasing central drive in the cat. These studies support the idea that reflexes are modulated in human locomotion. However, they leave open the key question of whether such modulation could functionally support speed control or gait transitions. It is experimentally currently unfeasible to study the interaction between reflexes and potentially emerging locomotor functions in isolation from other spinal circuits.

Neuromusculoskeletal models allow exploring the capabilities of reflex modulation in a controlled setting. Several previous models have successfully generated locomotion using CPG-based architectures or finite-state machines in combination with reflexes^20–25^. For example, the influential work of Geyer & Herr^20^ demonstrated that rhythmic walking could be generated using a reflex-based controller in combination with a finite-state machine, without requiring a CPG. This foundational work inspired a range of studies improving model stability^26–28^ or increasing its versatility^21,22,29–31^. Prochazka et al.^18^ also showed that adding stretch reflexes on top of centrally generated gait can modestly increase gait speed in the cat. However, they emphasize that larger speed changes require higher-level control strategies.

Most existing models rely on fixed controller parameters optimized for a specific gait and speed. Only a few studies have systematically examined whether locomotion can be modulated through changes in spinal reflex parameters alone. Existing works on speed modulation often rely on CPGs or high-level adaptation mechanisms^21–25,30,32–34^ typically addressing walking or running separately. Wang et al.^22^ obtained running and walking with the same model at various speeds but employed an offline re-optimization of parameters for every speed rather than continuous online modulation. The same applies for Song & Geyer^21^ who showed that their controller can achieve different walking speeds and unstable running. Most works focus on offline speed modulation, where a desired target speed is set before simulation and the parameters and locomotion speed stay constant during the simulation. An online speed modulation dynamically adjusting speed during run-time has been investigated in some works^25,34–36^. However, these approaches either rely on switching between a limited set of preoptimized parameter configurations, thereby enabling only discrete speed levels, or achieve speed modulation by adapting supraspinal modules or central pattern generator components. Recently, Koseki et al.^37^ devised a modulation strategy for the controller of Wang et al.^22^. Their approach allows to continuously adapt the walking speed by adapting all controller parameters which include force and length feedback as well as muscle-driven proportional derivative control of joint angles. While these are promising results they are adapting highly fused signals like target joint angles. Also all works implementing online speed modulation focus on one gait only (walking or running). As a result, the potential of reflex modulation for speed control across human gaits remains under-explored.

In recent work, we introduced a reflex controller based purely on length and force feedback^38^. This architecture demonstrated that a wide range of gaits—including walking, running, and hopping—could emerge from a compact set of reflexes. However, the model used fixed parameters for each solution and did not address how reflexes might be modulated to achieve speed or gait transitions. In this study, we address this gap, by adding a reflex modulation strategy to the controller. We then investigate whether modulation of reflex gains and offsets alone—without rhythm generators or high-level state machines—can enable online and offline control of locomotion speed across different gaits. Not excluding a potential role of CPGs like in Dzeladini et al.^30^ and Di Russo et al.^31^, we can show that the modulation of reflex gains and offsets is, in principle, enough to allow for a wide speed range for both walking and running.

This work investigates the potential of spinal reflexes to support versatile locomotion. Within the complex interplay of the different components of the central nervous system, basic locomotor functions could be taken-over by reflexes reducing the computational load for higher levels. Our study is based on prior findings that reflex gains are subject to modulation. While the origin of this modulation is not modeled—whether from supraspinal commands, CPGs, or other neural mechanisms—it aims to isolate and understand what reflexes themselves could plausibly contribute, assuming such modulation exists. In doing so, the results contribute to the ongoing debate about the roles of reflexes, CPGs, and higher-level control in human locomotion, offering insights relevant to both neuroscience and the development of bioinspired locomotion controllers.

## Results

This study investigates what spinal reflex mechanisms alone can achieve in modulating human locomotor speed in walking and running as well as in generating gait transitions. To address this question, we used a neuromusculoskeletal simulation controlled by a reflex controller based on fixed-gain muscle length and force feedback as proposed in our previous work^38^. In the context of this model, “reflexes” refer to simplified spinal feedback pathways in which muscle length or force signals are processed through a gain and offset and then provide excitatory or inhibitory input to the same or another muscle.

Our approach begins from a baseline gait and optimizes the 71 parameters (i.e., the reflex gains and constant offsets) to produce stable walking (*w*) or running (*r*) at different speeds (subsection Optimization). This dataset was then used to identify a subset of key reflex parameters, i.e. reflex pathways most influential for modulating speed in each solution. A new optimization only varying the key parameters generated a new dataset. Based on nonlinear regression on this dataset, a speed modulation function was devised adapting the key parameters to obtain a target speed $v_\text {tgt}$. Here, $v_\text {tgt}$ represents an abstract desired velocity input to the controller rather than a specific physiological variable; its biological origin is not modeled in this study. The speed modulation function was evaluated both in preset (subsection Offline modulation) and dynamic (subsection Online modulation) control scenarios. In the online condition, we further examined whether abrupt switching of reflex parameters could produce smooth transitions between walking and running. The results of the pipeline were compared starting from two different walking (${\bf p}_{w}$ and ${\bf p}_{w,alt}$) and running (${\bf p}_{r}$ and ${\bf p}_{r,alt}$) baseline gaits (subsection Analysis of speed modulation function). Detailed descriptions of the model and optimization procedures are provided in the Methods section.

### Optimization

**Table 1:** Overview speed ranges. Speed range $v_\text {range}$ and minimum/maximum speed ($v_\text {min}$/$v_\text {max}$) for optimization using all parameters (Opt 71), optimization using 30 parameters (Opt 30), and modulation using the identified 30 key parameters and a nonlinear regression of degree 3 (Mod 30). Results are given starting from the main walking set ${\bf p}_{w}$ and the main running parameter set ${\bf p}_{r}$.

	Walk init ${\bf p}_{w}$			Run init ${\bf p}_{r}$		
(m/s)	Opt 71	Opt 30	Mod 30	Opt 71	Opt 30	Mod 30
$v_\text {range}$	1.48	1.25	0.75	1.40	1.36	0.76
$v_\text {min}$	0.45	0.51	0.69	2.00	2.02	2.39
$v_\text {max}$	1.93	1.76	1.44	3.40	3.38	3.16

Our speed optimization resulted in wide velocity ranges for both walking and running. The obtained walking parameter set covered speeds ranging from 0.45 m/s to 1.93 m/s, and the running parameter sets from 2.0 to 3.4 m/s, corresponding to ranges of 1.48 m/s and 1.40 m/s respectively, as summarized in Table 1.

Optimizations with different numbers of key parameters ($n={5} \text { to } {30}$) were run as a next step. For walking and running, $n=10$ parameters already allowed for a speed range of 0.83 m/s and 0.95 m/s respectively, but unsurprisingly increasing the numbers of key parameters allows for a further range increase (0.8 m/s to 1.48 m/s (walking) and 0.38 m/s to 1.40 m/s (running) for increasing number of key parameters from 5 to 71, compare supplementary material Table S1). With a subset of 30 key parameters, similarly wide velocity ranges as with the optimization including all 71 parameters, both for walking and running, were found, (walking: 1.25 m/s and running: 1.36 m/s, as indicated in Table 1).

### Offline modulation

**Fig. 1:**
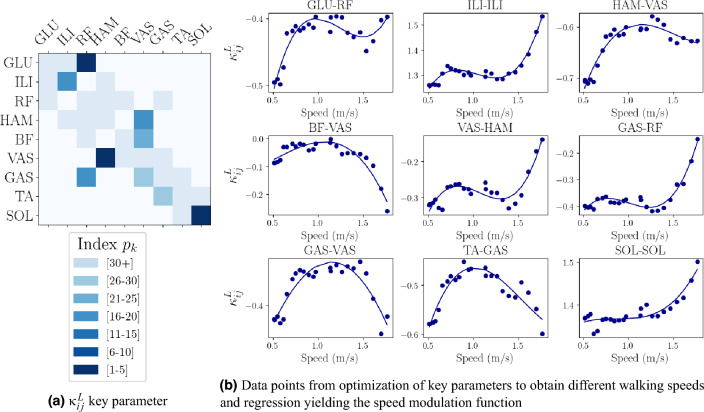
Modulation strategy. Exemplary results of the key parameter determination and modulation strategy for Walk Init ${\bf p}_{w}$. (**a**) All length reflexes of the parameter set using 30 key parameters are shown, colored based on their index in the first principal component. (**b**) The data set for each of these key reflexes over speed, together with the fitted regression curve of degree 3 are given. Note the different y-axes scales. Muscle acronyms: *GLU* gluteus, *ILI* iliopsoas, *RF* rectus femoris, *HAM* hamstrings, *BF* biceps femoris short head, *VAS* vastus, *GAS* gastrocnemius, *TA* tibialis anterior, *SOL* soleus.

The nonlinear regression strategy of degree 1 to 3 (described in Methods) was tested for its effectiveness in achieving stable speed modulation for varying numbers of key parameters. Overall, the proposed speed modulation function proved successful and could generate parameter sets of stable walking and running at considerable speed ranges (see Table 1 and supplementary material Table S1). Considering walking and running, the modulation strategy with 30 key parameters and a degree of 3 showed the overall best results. Figure 1 exemplarily shows the key length parameters for walking and their values, as well as the regression. All 30 key parameters over speed for Walk Init ${\bf p}_{w}$ and their approximation with the chosen modulation strategy are displayed in the supplementary material Fig. S1. With this modulation strategy, the speed for walking could be modulated in a range of 0.65 m/s, and for running in a range of 0.76 m/s (see also Table 1). We used this setting for all further analysis.

**Fig. 2:**
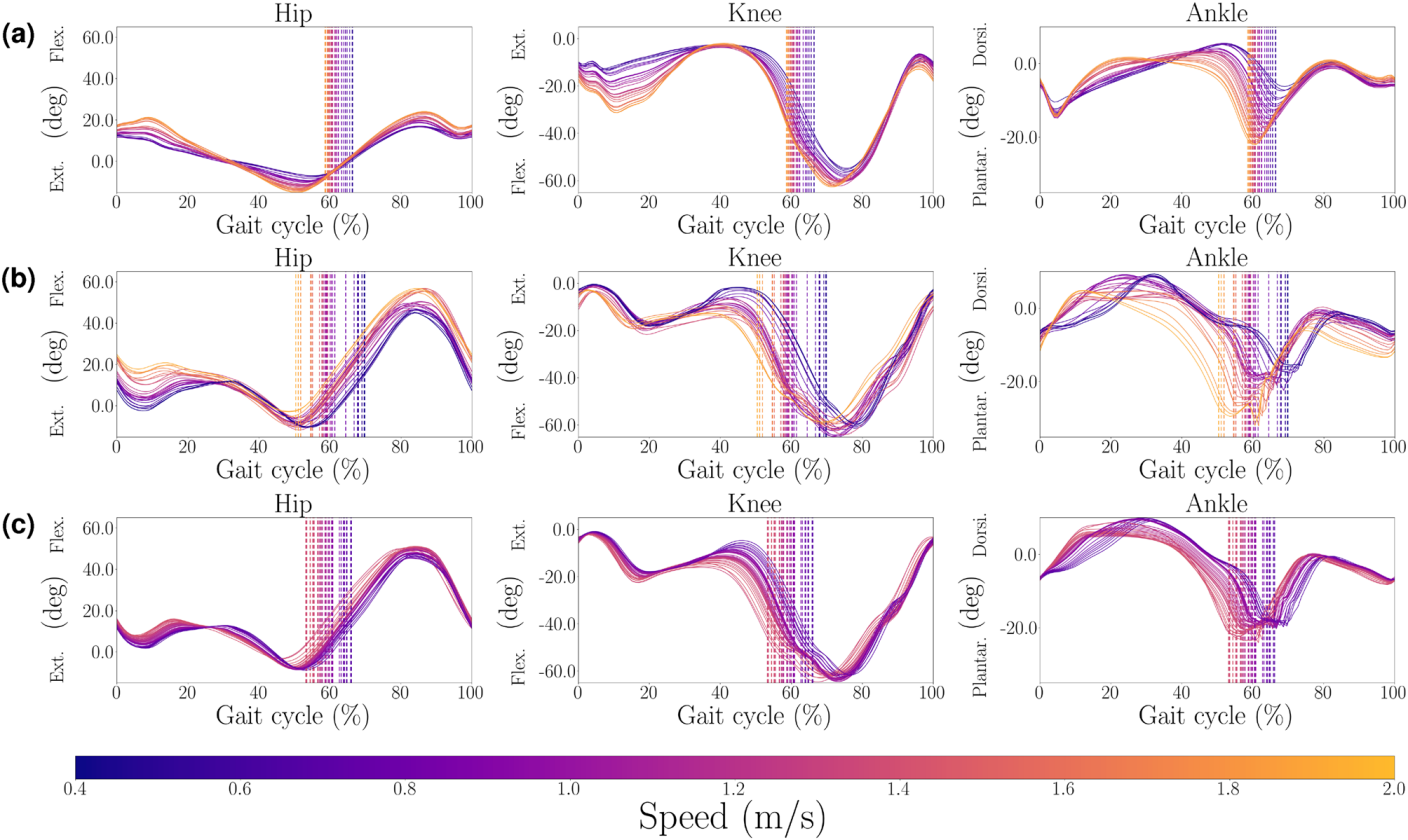
Joint angles during walking. Hip, knee, and ankle angles at varying walking speeds: (**a**) Experimental data of walking^39^, 0.5 m/s to 1.85 m/s, (**b**) Walk Init ${\bf p}_{w}$: optimization with 71 parameters, 0.45 m/s to 1.93 m/s, (**c**) Walk Init ${\bf p}_{w}$: modulation with 30 parameters, 0.69 m/s to 1.44 m/s. Dotted vertical lines indicate toe-off. The zero configuration represents standing position. Mean RMS values and standard deviation for comparison of experimental data to optimization: hip (16.30±1.79)$^{\circ }$, knee (8.69±1.49)$^{\circ }$, ankle (6.54±0.89)$^{\circ }$.

The kinematic changes over speed are displayed in Figs. 2 (walking) and 3 (running) for experimental data^39,40^, optimization with all 71 parameters, and modulation based on 30 key parameters and a regression of degree 3. In both experimental and simulation data, the stance phase is shortened with higher speeds. Changes are relatively continuous with speed. While not all trends are replicated, several trends are similar (e.g., higher ankle plantar flexion with higher speeds). The simulation results for running show less pronounced differences between speeds than in walking. The muscular activations for walking and running compared to experimental data are provided in the supplementary material Figs. S2 and S3. Overall, they show continuous shapes without abrupt changes.

**Fig. 3:**
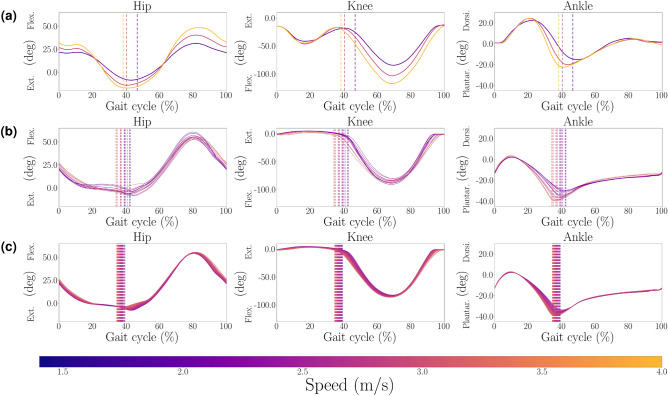
Joint angles during running. Speed modulation during running : (**a**) Experimental data of running at 2 m/s, 3 m/s and 4 m/s^40^. (**b**) Run Init ${\bf p}_{r}$: optimization with 71 parameters, 2.0 m/s to 3.40 m/s. (**c**) Run Init ${\bf p}_{r}$: modulation with 30 parameters, 2.39 m/s to 3.16 m/s. Dotted vertical lines indicate toe-off. The zero configuration represents standing position. Mean RMS values and standard deviation for comparison of experimental data to optimization: hip (13.23±2.38)$^{\circ }$, knee (24.15±1.49)$^{\circ }$, ankle (20.12±0.76)$^{\circ }$.

**Fig. 4:**
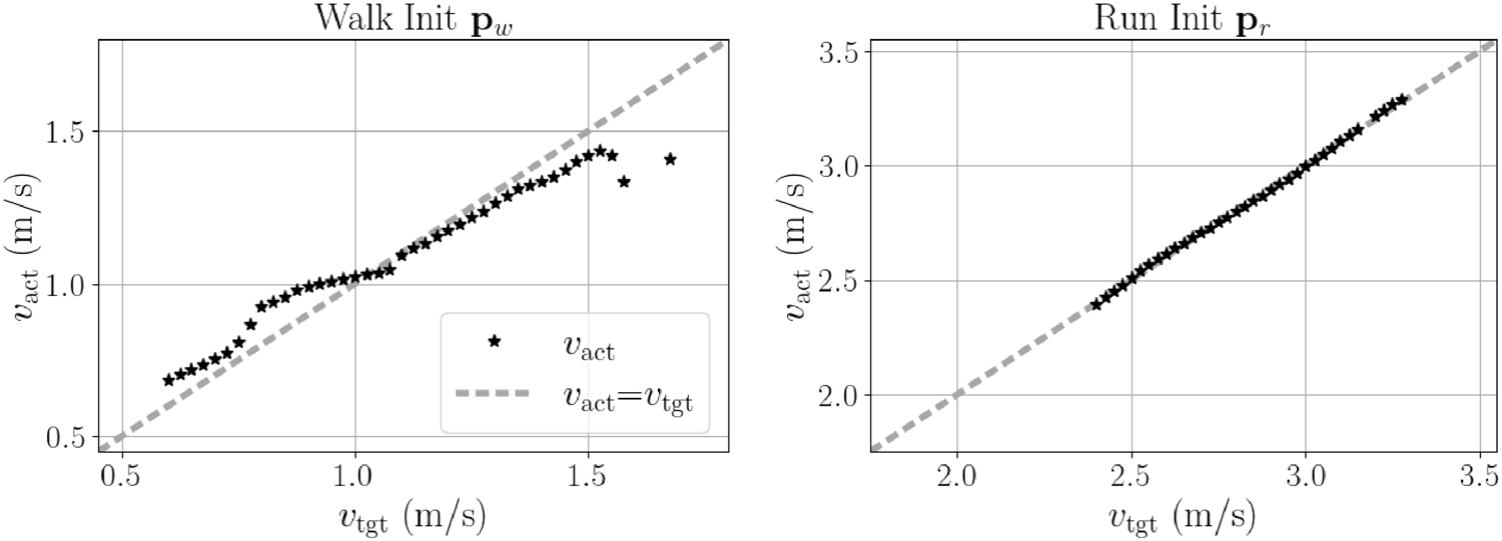
Offline modulation. Actual velocity $v_\text {act}$ versus target velocity $v_\text {tgt}$ (black) for the modulation strategy with 30 parameters and deg 3 for walking (left) and running (right). $v_\text {act}$ is only calculated for stable solutions with $d_\text {sim}$ = 50 s.

Additionally, a more in-depth analysis and testing of the speed modulation strategy was performed. While the overall speed range indicates the modulation’s validity, the relationship between target velocity $v_\text {tgt}$ and reached velocity $v_\text {act}$ was also evaluated. Except for the highest stable target velocities in walking, $v_\text {act}$ is monotonically increasing with increasing $v_\text {tgt}$ both for walking and running. The effective speed $v_\text {act}$ reflects the target speed very well for the running modulation and is very close to the ideal linear relationship (running panel Fig. 4), whereas for walking the relationship is not as linear, and the actual velocity exceeds the target velocity for low velocities and undershoots for high velocities (walking panel Fig. 4).

### Online modulation

**Fig. 5:**
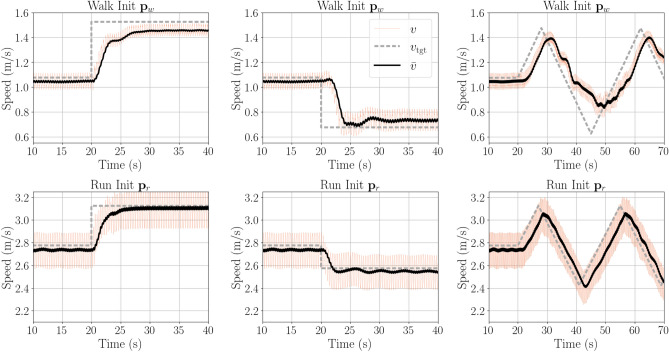
Online modulation. The prescribed target velocity $v_\text {tgt}$ (grey), torso velocity *v* (orange) and average velocity $\bar{v}$ of the torso (calculated with a moving average of 1 s, black) for walking (top) and running (bottom) for maximal (left) / minimal (center) steps and ramps (right) within the stable modulation speed range. Walking does not reach the target velocity due to the non-strictly linear relationship of $v_\text {tgt}$ and $v_\text {act}$ (see Fig. 4).

In order to test online speed modulation, the speed modulation function was used dynamically such that the controller parameter values change during run-time (for more details, refer to Methods). The results show that the found stable range of [$v_{\text {tgt,min}},v_{\text {tgt,max}}$] can indeed be used to dynamically accelerate and decelerate the model during running and walking by modulating the 30 key reflexes online (see Fig. 5). Both, the walking and running modulation strategy, allow for relatively large speed increases (steps of $v_\text {tgt}$ of 0.45 m/s walking and 0.35 m/s running). Walking does not exactly reach the target velocity. For steps decelerating the model, the walking strategy allows steps of 0.4 m/s, while the running strategy gets destabilized for steps greater than 0.2 m/s. The steps do not visibly disturb the gait and are not timed to a specific point in the gait cycle. We provide a video of the minimum and maximum step for walking and running as supplementary material (Video V1). Furthermore, both strategies track ascending and descending speed ramps successfully.

Furthermore, an abrupt change of all controller parameters allowed for gait transitions between walking and running. For this, the initial optimization’s fast walking and slow running solutions were used as starting values for an additional optimization where at specific points all controller parameters were changed (e.g. from walking to running, see Methods for more details). The optimization indeed found parameter sets allowing for a stable sequence of walk-run-run-walk transitions by abruptly switching between different parameter sets. A video of the optimization result is provided as supplementary material (Video V2).

### Analysis of speed modulation function

Additional tests using different initial parameter sets were conducted to test how much the presented results depend on the initial chosen solution. Namely, one additional walking initialization ${\bf p}_{w,alt}$ and running initialization ${\bf p}_{r,alt}$ were evaluated (see supplementary material, section Additional initial parameter sets for the results). These initial parameter sets were chosen explicitly to differ in parameter space, but also kinematically (walking) or in terms of gait speed (running) (see also supplementary Fig. S4 showing the difference in the initial parameter sets of the two walking conditions and the two running conditions). The resulting joint angle time series at different speeds are presented in Figs. S5 and S6. These additional tests resulted in similarly effective speed modulation, offline and online, demonstrating that the method adapts across different conditions (compare also supplementary material Table S2, Figs. S7 and S8). The determined key reflexes vary markedly across the different solutions. For instance, for walking, ${\bf p}_{w,alt}$ relied more on length-based reflexes, while ${\bf p}_{w}$ depended more on force-based reflexes (${\bf p}_{w}$: 9 KL, 16 KF, ${\bf p}_{w,alt}$: 17 KL, 9 KF). The only reflex that is part of the determined key reflexes for all four cases is the monosynaptic length reflex of the SOL muscle. All sets of key reflexes contain at least one reflex targeting each of the nine muscles (see Fig. S9). The initial parameter sets differ in which muscle receives a high number of reflexes, e.g., GAS receives a relatively high number of reflexes for ${\bf p}_{w}$ and ${\bf p}_{r}$, whereas ILI and VAS are most prominent for ${\bf p}_{r,alt}$ and RF and TA for ${\bf p}_{w,alt}$. All types of parameters (length, force, constant offsets) are present for all four modulation strategies (see Fig. S9). For a more comprehensive comparison see supplementary Figs. S9 and S10.

Speed modulation may be achievable with fewer key parameters. Testing our approach with varying numbers of key parameters, revealed that considerable speed ranges can be obtained with fewer key parameters in both optimization and modulation (e.g. walking range for 5 key parameters: 0.8 m/s, modulation range for running, regression degree 3: 0.95 m/s, see also Table S1).

## Discussion

This study explores the capabilities of reflex modulation to allow for gait speed modulation and gait transitions in human. While experimentally it is difficult to isolate reflexes from other circuits in the spinal cord, simulation studies allow to probe their potential in taking over different locomotor function. This study demonstrates that reflexes alone can generate and modulate both human walking and running across a broad range of speeds. Most previous studies achieve speed changes either through CPG modulation, predefined state machines, or switching between a limited set of pre-optimized gaits^21–25,30,32,33^. Recently, Koseki et al.^37^ demonstrated continuous online speed modulation by adapting the full set of controller parameters of the model by Wang et al.^22^. However, their approach includes modulation of higher-level parameters like desired hip angles and was demonstrated for walking only. Our study extends this approach and our results show that continuous speed modulation in walking and running and walk–run transitions can emerge from modulation of spinal reflex gains alone. The number of reflex parameters which have to be adapted is reduced to a set of key parameters. Furthermore, the present study applies the same modulation strategy to achieve offline and online speed changes around different initial parameter sets. The results show that both preset (offline) and dynamic (online) speed control in walking and running is in principle possible based on reflex modulation.

### Reflex modulation allows for speed control and gait transitions

The results show that wide speed ranges and gait transitions can be achieved solely by modulating reflex gains and offsets, demonstrating that reflex pathways alone are sufficient to control locomotion speed across different gaits within the modeled conditions.

The approach builds on the controller proposed in Bunz et al.^38^, and extends it from single control parameter sets to the dynamic connection of different solutions. Entirely based on the modulation of reflex gains, the speed range of normal human walking can be covered^41^, and very slow speeds of minimally 0.45 m/s and very fast walking speeds of maximally 1.93 m/s can be reached. While the minimal running speed aligns well with human capabilities, the maximum speed (3.40 m/s) is below the maximal speed of human running (running speed range of 2 m/s to 6 m/s^42^). However, it is close to the preferred running speed of experienced runners (3.7 m/s^43^) and the maximum speed of untrained persons (3.88 m/s^44^). Increasing maximal muscle forces to reflect experienced runners^45^ or model complexity (e.g., adding arms^46^) could likely increase the maximum velocity of the model. Remarkably, the superior and inferior limits of the speed optimization for running and walking reflect the average walk-to-run transition speed of around 2.0 m/s. Importantly, these speed limits were not hard-coded into the model but emerged naturally from optimizing the reflex gains.

Notably, the results indicate that not all parameters require re-optimization. A subset of key reflexes is sufficient, reducing the number of reflex loops in the spinal cord that descending pathways must to project to. Optimizing only 30 parameters is sufficient to reach almost the same speed ranges as optimizing all 71 parameters. Nonlinear regression can approximate these parameters for a given target speed, allowing for flexible, reflex-based speed modulation. Simpler than complete optimization, this strategy covers normal human walking speeds and running speeds up to 3.31 m/s.

The proposed approach can also extend to gait transitions. The preliminary results indicate that transitions between walking and running are feasible through abrupt parameter changes. While a more in-depth analysis on transitions remains to be performed in future works, these results show that the reflex controller allows for smooth transitions between walking and running. The presented speed modulation results will, therefore, provide a valuable starting point to study gait transitions. However, more detailed investigations are needed to determine the optimal timing within the gait cycle and the similarity of parameter sets required for stable transitions. The current approach of abruptly changing all controller parameters was adopted for the proof-of-concept of reflex based transitions but in humans smoother transitions are more likely to happen^47^. Gait transitions, therefore, remain a promising area for future research, potentially allowing for more natural, continuous transitions without the need for abrupt, global parameter shifts.

### Characteristics of the modulation

For running, the modulation strategy leads to an almost perfect correspondence between target speed and effective speed, even though the modulation strategy is completely open loop. For walking, the correspondence is not entirely linear pointing to the fact that some parameters might still be missing. Still, the resulting gaits of the optimization show comparatively continuous changes in joint angles and muscular activations, demonstrating that different speeds seem to be continuously connected in kinematic space.

Inspection of the parameter sets obtained from optimization of key reflex parameters suggests that the modulation of reflex parameters changes gradually with speed, as adjacent solutions show continuous shifts in parameter values that can be approximated by nonlinear regression. Tests with four distinct initial parameter sets (two walking ${\bf p}_{w}$, ${\bf p}_{w,alt}$ and two running ${\bf p}_{r}$, ${\bf p}_{r,alt}$) show that speed can be modulated across all cases, with notable variation in the utilized key reflexes. Each strategy uses a mix of length, force, and offset parameters, but their relative contributions vary. However, the modulation around ${\bf p}_{w}$ relies more on force feedback whereas for ${\bf p}_{w,alt}$ more length reflexes are involved. Also, for all strategies, at least one parameter targeting each muscle is present, but each set of key reflexes relies on different muscles. Overall, this clearly shows that the found strategies differ markedly. A common element across all sets is the monosynaptic length reflex of soleus, aligning with experimental findings that highlight the prominent role of a modulation of the soleus stretch reflex for speed modulation^48,49^. This diversity in strategies mirrors the variability observed in human muscle activity patterns^3,50^ and suggests that the controller captures some of the inherent flexibility of human gaits.

Except for very fast walking (${\bf p}_{w}$), the modulation strategy produces a monotonic function between $v_\text {tgt}$ and $v_\text {act}$ for all tested initial parameter sets. This paves the way for the extension of the modulation to an online setting. Indeed, it tracks both abrupt and gradual speed changes effectively. Due to the non-strictly linear correspondence of $v_\text {tgt}$ and $v_\text {act}$ for walking, it does not precisely follow the prescribed speed curve, but it tracks the shape both for steps up and down and for continuous speed changes along a ramp. This also applies for running where the prescribed speed in addition is tracked well. The abrupt speed changes show some delay and are not fast enough to reflect maximal speed changes in humans. Here, the abrupt speed change represents a change in control input (desired speed), not an external perturbation. The slower response therefore reflects adaptation dynamics rather than delayed reflexive reactions.

It is important to consider that the controller has to maintain stability based solely on reflexes, rather than relying on a more sophisticated balance control such as vestibular feedback. In this context, stability refers to the model’s ability to maintain coordinated gait patterns across different speeds without falling. Indeed, stability emerges inherently from the reflex architecture, whereas most other models include additional PD controllers^20–22,29^. Other models also present passive dynamics or preflexes that handle stability over the gait cycle^51,52^. In the present model, the stability may be the key limiting factor to broader velocity ranges. The results for the additional running initial parameter set (Run Init ${\bf p}_{r,alt}$) underline this reasoning. While the parameter sets between 2.925 m/s to 3.1 m/s do not lead to stable gait, higher velocities between $v_\text {tgt}$= 3.125 m/s to 3.175 m/s generate stable running with $v_\text {act}$ close to $v_\text {tgt}$ (compare supplementary material Fig. S7). During optimization, parameter adjustments can ensure stable gait, while the modulation strategy neglects this additional stabilization constraint and simply approximates the relationship between parameter values and resulting speed. Therefore, it is already remarkable that stable gait in such a vast range is possible using this simple modulation strategy, which introduces considerable noise into the controller.

### Limitations

The generic approach used here, aimed to support speed modulation around multiple initial parameter sets. The optimization results show that 10 parameters already allow to obtain parameter sets at different speeds (walking range: 0.83 m/s, running range: 0.95 m/s), and even with only five parameters, the optimization yields a speed range of 0.8 m/s for walking and 0.38 m/s for running. The setting of 30 parameters and a regression of degree 3 was chosen as a trade-off to allow generalization of the approach to different initial parameter sets while reducing the number of used parameters. Although 30 parameters might seem a lot, they are not fully independent as they are interconnected and bound to sensory feedback, limiting the risk of overfitting. Furthermore, the determination of the parameter sets is based on the results of two optimizations (minimizing/ maximizing velocity). However, the speed modulation strategies could differ for the two optimizations. Indeed, the correspondence between $v_\text {tgt}$ and $v_\text {act}$ of the main walking parameter does not follow a single function but rather shows two distinct patterns, separated at approximately the initial velocity $v_\text {init}$= 1.1 m/s (see Fig. 4). Although the methodology thus introduces considerable noise, the modulation strategy works well and allows for stable gait generation in a considerable speed range. Repeating the study with multiple initial conditions could help identify a larger spectrum of possible solutions and provide further insights into the global role of the reflex parameters. Conducting such an analysis constitutes a complete study on its own, requiring comprehensive exploration and evaluation, and is therefore left for future work. As argued above, the generic approach of this study proved sufficient to demonstrate the potential of reflex-based speed modulation.

As this work focused on the role of reflexes in speed modulation and gait transition, the model was intentionally kept anatomically and neurologically simplified. Also, the optimization process based on high-level objectives was not intended to represent a biologically plausible learning mechanism, but rather a technical optimization procedure to systematically explore the control potential of reflex modulation. Future studies could incorporate vestibular feedback, 3D extensions, addition of arms, more complex control hierarchies or a biologically plausible learning algorithm. While our study used a technical, regression-based approach for reflex gain modulation, alternative strategies like CPGs or muscle synergies, potentially informed by PCA, could provide a more biologically plausible framework. Reflexes alone may not fully account for speed modulation, as mechanisms like CPGs likely play a complementary role in human locomotion^9^. Indeed, in previous work in cats, a single input to the CPG could both change the speed and automatically modulate stretch reflexes^19^, illustrating how CPGs can simplify speed control relative to the mechanism proposed in this study. Nevertheless, reflex modulation is a well-established mechanism in biology, occurring throughout the gait cycle, and our results demonstrate that reflexes can provide the core components for flexible speed control. In practice, this suggests that reflexes could reduce the computational load on higher-level structures, while CPGs could efficiently provide baseline rhythm generation and facilitate broader speed ranges with fewer control parameters. Importantly, the present results demonstrate that reflex modulation is sufficient in principle to generate flexible speed control within the model, but they do not imply that human neural control relies exclusively on the same mechanisms.

## Conclusion

These findings underline the potential of reflex-based control for speed modulation and gait transitions, suggesting that reflexes could significantly reduce the computational burden on higher-level components. Potential neural substrates handling reflex modulation could be supraspinal commands, CPGs, or circuits mediating muscle synergies. This work sets the stage for future studies eventually allowing to devise a reflex-based controller for bipedal locomotion that is capable of not only speed modulation but also gait transitions and possibly even goal-directed movements.

## Methods

**Fig. 6:**
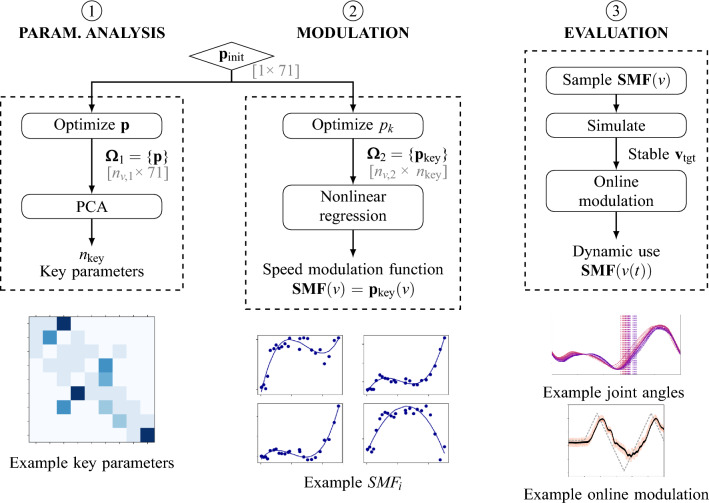
Methodology. Overview of the proposed pipeline to study speed modulation and derive a reflex-based speed modulation strategy. (1) Starting from an initial parameter set ${\bf p}_{init}$, optimizations yield a data set $\boldsymbol{\Omega }_1$ of parameter sets at $n_{v,1}$ different speeds. A principal component analysis (PCA) on $\boldsymbol{\Omega }_1$ is used to determine key parameters. (2) Another set of optimizations allowing a variation of only $n_\text {key}$ key parameters $p_k$ is run. The resulting new data set $\boldsymbol{\Omega }_2$ of parameter sets at $n_{v,2}$ different speeds is then used to derive a modulation strategy that approximates the relationship of each key parameter with locomotion speed using nonlinear regression. (3) The derived strategy is then tested offline (setting a desired target speed before simulation) and online (dynamically adjusting speed during run-time).

The aim of this work is to study reflex-based speed modulation in human neuromusculoskeletal models. For this, we use our recently proposed reflex-controller^38^. To study speed modulation, the parameters of the controller are optimized to find the possible speed range for walking and running. The optimization results are then used for tests on gait transitions. Furthermore, parameter sets at different speeds are extracted from the optimization results. This data is used to determine the key parameters modulating speed. Their relationship with speed is approximated using nonlinear regression, and the capabilities of this speed modulation strategy are tested in both offline and online settings. The used model and controller as well as the mentioned steps are detailed below, and visually summarized in Fig. 6.

### Model and controller

#### Model

**Fig. 7:**
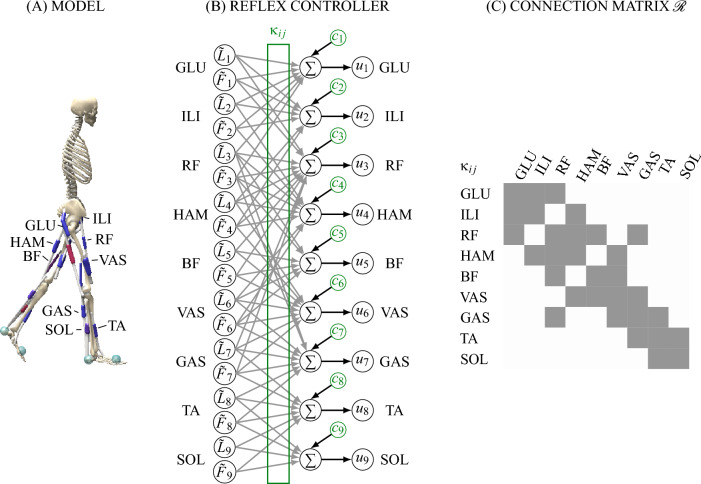
Controller architecture. Speed modulation is studied within the reflex controller proposed in Bunz et al.^38^, which can produce running and walking in a human musculoskeletal model. (**A**) The planar musculoskeletal model consists of seven segments connected by six joints, which are actuated by 18 Hill-type muscles. (**B**) The reflex controller calculates stimulation for each muscle *i* from delayed force $\tilde{F}$ and length $\tilde{L}$ feedback with gains $\kappa _{ij}$ and a constant offset $c_i$. Feedback gains and offsets (highlighted in green) are optimized based on high-level objectives. (**C**) Implemented pathways include homonymous and antagonistic pathways for length and force (gray).

In this work, we use a planar model consisting of seven segments (torso and two legs with femur, tibia, foot) actuated by 9 Hill-type musculotendon units (MTU) per leg, as detailed in Bunz et al.^38^. The model represents an adult human male of 74.5 kg and 1.80 m height. The modeled MTUs are gluteus (GLU), iliopsoas (ILI), rectus femoris (RF), hamstrings (HAM), biceps femoris short head (BF), vastus (VAS), gastrocnemius (GAS), tibialis anterior (TA) and soleus (SOL) (see Fig. 7). These MTUs generate the actuation of the three joints per leg (hip, knee and ankle). For more information on the model see Bunz et al.^38^.

#### Controller

The stimulation $u_i (t)$ of each MTU *i* is calculated using the reflex controller proposed in our previous work^38^. In short, the controller consists of a network of constant-gain ($\kappa$) delayed ($\Delta t$) length ($\tilde{L}$) and force ($\tilde{F}$) reflexes directly connecting the sensory information to muscle stimulation (see also Fig. 7):

1 $$\begin{aligned} u_i (t) = c_i + \sum _{j \in \mathcal {R}_i}^{}[\kappa _{ij}^L \tilde{L}_j (t- \Delta t)+\kappa _{ij}^F \tilde{F}_j (t- \Delta t)] \end{aligned}$$

Two types of connections are included in the connection matrix $\mathcal {R}$: *homonymous* connections, where the muscle is stimulated by its own feedback and *antagonistic* connections, where the muscle receives stimulation through the feedback of one of its antagonists. With the nine muscles of each leg, this leads to 31 possible connections. Additionally, for each muscle there is a constant offset $c_\text {i}$. In total, the controller therefore contains 71 free parameters (31 $\kappa ^L$ and $\kappa ^F$ and 9 *c*). The control is symmetric for both legs and the same control parameters are used. The model and controller are implemented in SCONE^53^ and Hyfydy^54^.

### Speed optimization

The reflex controller proposed in Bunz et al.^38^ is capable of generating various gaits. This work investigates the controller’s capabilities in terms of speed modulation, focusing on walking and running. To allow finding a modulation strategy that is continuous in parameter space, the methodology adopted in this study is based on a local search around parameter sets known to produce walking or running. Namely, as a first step to determine the speed range the controller can achieve, a local search for different velocities around a known solution for walking or running is performed. Start from a parameter set found to produce walking or running in our previous work^38^ , an optimization for minimum or maximum average velocity *v* is performed. Due to the start from a parameter set producing stable forward movement, speed minimization does not lead to a backward movement, but to a slower forward movement. More in detail, the optimization minimizes the cost function *J* containing a fall penalty and a velocity reward:

2 $$\begin{aligned} J= 100 \cdot J_{\text {vel}} + 100 \cdot J_{\text {fall}} \end{aligned}$$

with

3 $$\begin{aligned} J_{\text {vel}} = {\left\{ \begin{array}{ll} \frac{d_\text {sim}}{t_\text {max}} & \text {if minimize }v\\ - \frac{d_\text {sim}}{t_\text {max}} & \text {if maximize }v \end{array}\right. } \end{aligned}$$

and

4 $$\begin{aligned} J_{\text {fall}} = 1 - \frac{t_\text {sim}}{t_\text {max}} \end{aligned}$$

Here, $d_\text {sim}$ is the completed distance, which corresponds to the horizontal displacement of the center of mass (CoM) of the segment that, at the end of the simulation ($t_\text {sim}$), has the minimal/ maximal distance to the origin (for speed maximization/ minimization respectively). For speed minimization, $J_\text {vel}$ is simply the model’s average speed, i.e., it is zero if the model does not move and increases the faster the model locomotes. For speed maximization, it is the negated average speed, and as such, it gets more negative the faster the model locomotes. $J_\text {fall} = 0$ for solutions that do not fall within $t_\text {max}$ and increases until $J_\text {fall} = 1$ the earlier the model falls. Minimizing *J* thus yields stable solutions of forward walking/ running at slow or fast speeds. The maximum simulation time $t_{\text {max}}$ is set to 50 s and the simulation is stopped if the CoM of the model falls below 0.9 m. The Covariance Matrix Adaptation Evolution Strategy (CMA-ES)^55^ is used for optimization, and 20 optimizations with different random seeds are run for each initial parameter set. The standard deviation of the CMA-ES algorithm is set to 0.01 for all parameters, and the model’s initial states (torso and joint angles and velocities) are the same as those in our previous work^38^. Optimizations are terminated when the average relative improvement over the last 500 iterations is less than $10^{-5}$ per iteration.

This approach finds parameter sets leading to speed changes locally around the initial parameter set. In the following, the results for optimizations starting from one main walking and one running parameter set ${\bf p}_{w}$ and ${\bf p}_{r}$ are analyzed. The supplementary material contains additional results for one alternative initial parameter set for walking ${\bf p}_{w, alt}$ and running ${\bf p}_{r, alt}$. These additional two parameter sets were chosen to differ from the main parameter set in parameter space. For the two walking sets ${\bf p}_{w}$ and ${\bf p}_{w, alt}$, there is also a marked difference in kinematic space (e.g., large knee extension vs. knee flexion at early stance). For running, the solutions are parameter sets generating running at two different velocities while differing less in their joint angle trajectories.

### Key parameter determination

Starting from an initial parameter set ${\bf p}_{init}$ at a certain speed, the optimization process progressively identifies intermediate solutions with increasingly lower cost function values. As the optimizations start from an initial parameter set producing stable walking or running, these intermediate optimization solutions typically produce stable movement at increasing or decreasing speed (depending on whether*v* is minimized or maximized). The optimization with the largest speed range is determined from the 20 optimization runs for minimum and maximum speed, and all intermediate optimization results that produce a stable gait for $t_\text {max}$, i.e., that do not fall within $t_\text {max}$, are extracted. This leads to a set of parameter values over speed $\boldsymbol{\Omega }_1 = \{{\bf p}\}$ containing parameter sets ${\bf p}$ at $n_{v,1}$ different velocities. The exact number $n_{v,1}$ depends on the optimization and the number of intermediate solutions that were found. Taking the intermediate solutions rather than optimizing for different target speeds indirectly enforces continuous changes in parameter space, as each solution represents a gradual adjustment toward the optimal solution. This approach prevents abrupt shifts in the parameter set and minimizes the noise the optimization introduces. Combining the found solutions for minimum and maximum speed, $\boldsymbol{\Omega }_1 = \{{\bf p}\}$ can be used to analyze the effect of the different parameters on speed.

A principal component analysis (PCA) is run on the dataset $\boldsymbol{\Omega }_1$ to determine key parameters, i.e. reflex pathways most influential for modulating speed in each solution. For each scenario, the first principal component (PC) separated the data well depending on the gait speed and explained around $50\%$ of the variance (compare also supplementary material Table S3). Therefore, the reflex parameters with the largest weights in the first PC were considered key parameters. To determine the necessary number of parameters and test whether they allow to modulate speed, the optimization was rerun by fixing all parameters to their value in the initial parameter set and allowing a variation of only the $n_\text {key}$ chosen key parameters, varying the number of key parameters $n \in \{5,10, 15, 20, 25, 30\}$.

### Speed modulation

The optimization approach allows the investigation of the general speed range the controller can achieve and gives some insights into parameters with a substantial impact on speed. However, speed modulation directly using the compiled set of parameters only allows to reach speeds present in the dataset and requires a look-up table. Also, the optimization approach likely introduces unwanted variation in the parameter values as it simultaneously adjusts many parameters, including those that may not be essential for speed modulation. Furthermore, the reflex-controller may support continuous variation in parameter space instead of transitions between discrete points. Therefore, a more abstract modulation strategy is devised by deriving a speed modulation function ${\bf SMF}(v)={\bf p}_{\text {mod}}(v)$ that computes a parameter set ${\bf p}_{\text {mod}}$ for any given target speed. For this, the relationship of each key controller parameter (determined as described in the previous section) with speed is approximated using polynomial regression (see Eq. 5). For any target speed $v_\text {tgt}$, the 71 controller parameters are then determined using non linear regression (for key parameters) or the initial parameter values (for all other parameters, see also Eq. 7). This approach heavily relies on an inherent robustness of the controller to parameter variations.

#### Offline modulation—sampling of ${\bf SMF}(v)$

Speed modulation using the polynomial regression strategy is first tested offline. For each dataset, the regression coefficients $\beta _{n,k}$ are computed for all key parameters $p_k$ testing various polynomial degrees ($d_\text {reg} \in [1,2,3]$), with $\beta _{n,k} = 0$, if $n > d_\text {reg}$ and the coefficients being recalculated for every considered $d_\text {reg}$.

5 $$\begin{aligned} SMF_k(v_\text {tgt})= \beta _{0,k} + \beta _{1,k} v_\text {tgt} + \beta _{2,k} v_\text {tgt}^2 + \beta _{3,k} v_\text {tgt}^3 \end{aligned}$$

For a range of target speeds ($v_\text {tgt} = [0.5, 3.5]$, steps of 0.025), parameter sets are generated using the initial parameter values for all fixed parameters and the value of the regression for the respective $v_\text {tgt}$:

6 $$\begin{aligned} {\bf p}_{\text {mod}}(v_\text {tgt}) = \begin{pmatrix} p_{\text {mod},1}(v_\text {tgt})\\ \vdots \\ p_{\text {mod},71}(v_\text {tgt})\\ \end{pmatrix} \end{aligned}$$

and

7 $$\begin{aligned} p_{\text {mod},i}(v_\text {tgt}) = {\left\{ \begin{array}{ll} SMF_i(v_\text {tgt}) & \text {if }p_i\text { key parameter}\\ p_{i,\text {init}} & \text {else.} \end{array}\right. } \end{aligned}$$

It is then evaluated which parameter sets ${\bf p}_{\text {mod}}(v_\text {tgt})$ lead to stable gait and at which velocity $v_\text {act}$.

#### Online modulation—usage of ${\bf SMF}(v(t))$

The best setting during offline modulation was $d_\text {reg} = 1$ and $n=30$ for ${\bf p}_{w}$, and $d_\text {reg} = 3$ and $n=15$ for ${\bf p}_{r}$. Considering both initial parameter sets ${\bf p}_{w}$ and ${\bf p}_{r}$, the overall best setting during offline modulation was a regression of $d_\text {reg} = 3$ and $n=30$ key parameters. This setting was used for the following analyses. To probe the robustness of the speed modulation strategy ${\bf SMF}(v)$, the found stable range of target velocities $v_\text {tgt}$ was used and the controller parameters were adapted accordingly during simulation. Namely, a set of target speeds $v_\text {tgt}$ was prescribed over time and ${\bf SMF}(v(t))$ was used dynamically to switch between the parameter sets ${\bf p}_{mod}(v_\text {tgt})$. For the time series of $v_\text {tgt}(t)$, abrupt steps in target velocity and ramps were tested. $v_\text {tgt}(t)$ always starts in the middle of the speed range with

8 $$\begin{aligned} v_{\text {tgt,start}} = v_{\text {tgt,min}}+ \frac{v_{\text {tgt,max}}-v_{\text {tgt,min}}}{2}. \end{aligned}$$

Here, $v_{\text {tgt,min}}$ and $v_{\text {tgt,max}}$ are the target speeds at which the actual velocity $v_{\text {act}}$ was minimal/ maximal while still yielding a movement without falling until $t_\text {max}$. In order to start in steady-state, the simulation was started with $v_{\text {tgt,start}}$ for 20 s. First, the response to steps of different sizes was tested by increasing the step size in steps $j\in \mathbb {N}$ of 0.025 and testing in both directions, i.e. increasing and decreasing the speed, such that

9 $$\begin{aligned} v_{\text {tgt,end}} = v_{\text {tgt,start}} \pm j\cdot 0.025, j \in [1,..,25] \end{aligned}$$

and

10 $$\begin{aligned} v_\text {tgt,step}(t) = {\left\{ \begin{array}{ll} v_{\text {tgt,start}} & \text {if } t < {20}\text {s}\\ v_{\text {tgt,end}} & \text {else} \end{array}\right. } \end{aligned}$$

The results show the response to the step leading to the largest stable speed increase or decrease. Second, the response to ramps covering the stable target speeds of the modulation strategy was tested. For this, $v_\text {tgt}(t)$ accelerates and decelerates between $v_{\text {tgt,min}}$ and $v_{\text {tgt,max}}$ by changing the target velocity by $v_a$=0.05 m/s every second:

11 $$\begin{aligned} v_{\text {tgt,ramp}}(t) = {\left\{ \begin{array}{ll} v_{\text {tgt,start}} & \text {if } t < 20 \\ v_{\text {tgt,min}} + v_a \left| \left( \left( t - \frac{T}{4} \right) \bmod T \right) - \frac{T}{2} \right| & \text {else } \end{array}\right. } \end{aligned}$$

with

12 $$\begin{aligned} T=\frac{2\cdot (v_{\text {tgt,max}}-v_{\text {tgt,min}})}{v_a}. \end{aligned}$$

$v_{\text {tgt,ramp}}$ starts with $v_{\text {tgt,start}}$ and then first increases $v_{\text {tgt}}$ to $v_{\text {tgt,max}}$ followed by a decrease to $v_{\text {tgt,min}}$. To avoid using target speeds close to instability, the slowest and fastest stable $v_{\text {tgt}}$ were excluded when determining $v_{\text {tgt,}\{\text {min,max}\}}$.

### Gait transitions

The initial optimization results are additionally used for a preliminary study on the possibility of reflex driven gait transitions by looking at transitions between walking and running. In particular, parameter sets of running and walking at similar speeds, i.e., parameter sets of fast walking and of slow running, are manually selected from the optimization results. These parameters are used in a simulation setting where after 10 s, an abrupt switch between the two parameter sets happens. For example, starting with a slow running solution, after 10 s, the parameter values are set to the fast walking parameter set. Without adaptation of the parameters, the model falls shortly after the transition. Therefore, an optimization is started from these initial values to optimize the $2 \cdot 71$ parameters, focusing on achieving $t_\text {sim}=t_\text {max}$. First, this optimization was run for walk-run and run-walk transitions separately. In an iterative process, the solutions of transitions from walking to running and vice versa were then combined to obtain a setting where after transitioning from walking to running, another abrupt transition to running and then back to walking is performed (with intervals of 10 s, see also supplementary material Fig. S11). Starting from these initial values, the then $4 \cdot 71$ parameters are re-optimized to achieve stable locomotion for all three transitions (walk-run-run-walk). With this manual approach, finding a running parameter set that allowed the transition both from and to walking directly was impossible, which is why the intermediate run-run transition was added. As the transition times were fixed this could be a matter of timing, but the exact mechanisms of the transitions must be investigated in further studies.

## Supplementary Information

Supplementary Information 1.

Supplementary Information 2.

Supplementary Information 3.

## Data Availability

All necessary files to reproduce our results are available at https://doi.org/10.18419/DARUS-4764.
