# Supplementary material for: Modulating reflexes enables speed control in simulated human walking and running
Source: Sci Rep. 2026 Apr 21;16:13028. doi: 10.1038/s41598-026-48509-z (PMC13100031; doi:10.1038/s41598-026-48509-z)
Supplement: Supplementary file 1 — Supplementary Information 1. [file 41598_2026_48509_MOESM1_ESM.pdf]

Supplementary material for Modulating reflexes enables speed control in simulated human walking and running

Elsa K. Bunz[1,*], Alice J. Bruel[2], Auke J. Ijspeert[2,+], and Syn Schmitt[1,+]

[1]Institute for Modelling and Simulation of Biomechanical Systems, University of Stuttgart, 70569 DE
[2]Biorobotics Laboratory, Ecole Polytechnique Federale de Lausanne, 1015 CH
[*]elsa.bunz@imsb.uni-stuttgart.de
[+]these authors contributed equally to this work

Modulation strategy

This section provides the data set $\mathbf{\Omega}_2$ together with the regression results used for the modulation starting from Walk Init $\mathbf{p}_w$ (Fig. S1). Furthermore, the muscular activations obtained for the seven muscles for which Camargo et al.[1] provides experimental data over walking speed are displayed in Fig. S3. Also an overview of the results for different number of key parameters and regression degrees is given (Table S1). All more in-depth analysis have been performed with a setting of 30 key parameters and a regression of degree 3.

Table S1. Optimization and modulation results Results for varying number of key parameters and varying regression degrees for the two main inits $\mathbf{p}_w$ and $\mathbf{p}_r$. The number of used key parameters is given in the columns and for each number of key parameters the obtained velocity range for optimization (Opt) as well as modulation (Mod) is given. Gray cells are also shown in the main manuscript.

	**Walk Init $\mathbf{p}_w$**						
v_{range} (m/s)	71	30	25	20	15	10	5
Opt	1.48	1.25	1.38	1.21	0.81	0.83	0.80
Mod ($d_{\text{reg}} = 1$)		0.84	0.43	0.35	0.63	0.21	0.55
Mod ($d_{\text{reg}} = 2$)		0.59	0.26	0.44	0.71	0.53	0.48
Mod ($d_{\text{reg}} = 3$)		0.75	0.19	0.27	0.49	0.42	0.23

	**Run Init $\mathbf{p}_r$**						
v_{range} (m/s)	71	30	25	20	15	10	5
Opt	1.40	1.36	1.30	1.33	1.20	0.95	0.38
Mod ($d_{\text{reg}} = 1$)		0.10	0.17	0.27	0.25	0.36	0.39
Mod ($d_{\text{reg}} = 2$)		0.68	0.87	0.75	0.89	0.71	0.20
Mod ($d_{\text{reg}} = 3$)		0.76	0.76	0.62	0.97	0.52	0.37

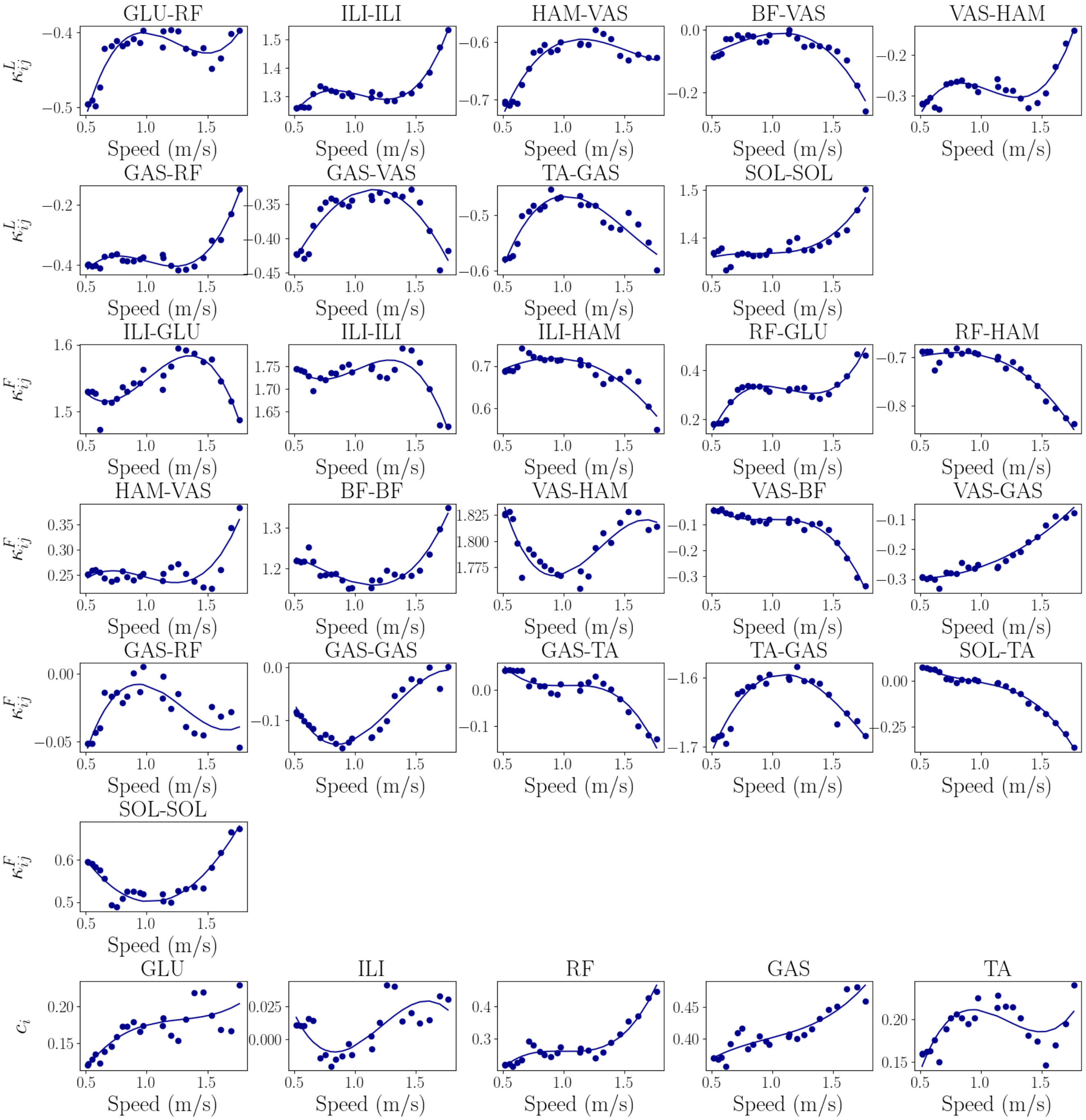

Figure S1. **Modulation strategy** Key parameters found for Walk Init $\mathbf{p}_w$, using the studied modulation strategy with 30 key parameters. The data set $\mathbf{\Omega}_2$ describing the relation of each key parameter with speed changes is displayed, together with the obtained regression results ($d_{\text{reg}} = 3$) which were used to modulate speed offline as well as online. Note the different y-axes scales.

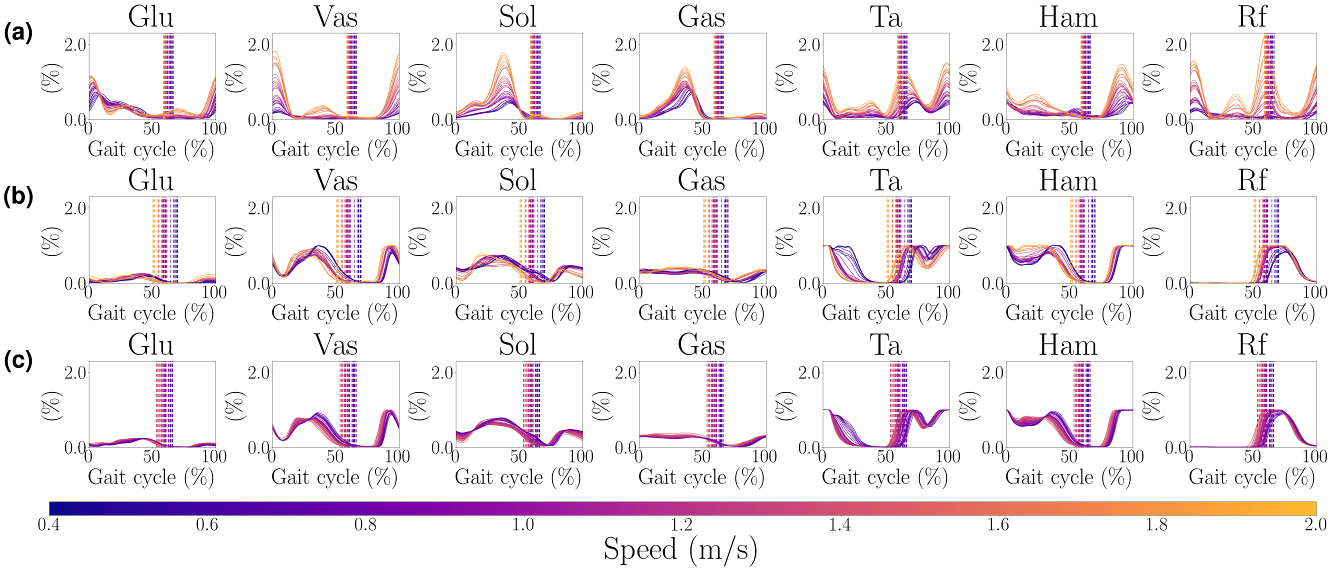

Figure S2. Muscular activations walking Muscular activation at different speeds in comparison for a) Experimental data from Camargo et al.[1] (0.5 m/s to 1.85 m/s) normalized for each subject with respect to the average amplitude at a speed of 1.35 m/s b) Optimization with 71 parameters starting from Walk Init $\mathbf{p}_w$ (0.45 m/s to 1.93 m/s) c) Modulation with 30 parameters (0.69 m/s to 1.44 m/s) starting from Walk Init $\mathbf{p}_w$. Only muscles where Camargo et al.[1] provides experimental data are shown. Dotted vertical lines indicate toe-off.

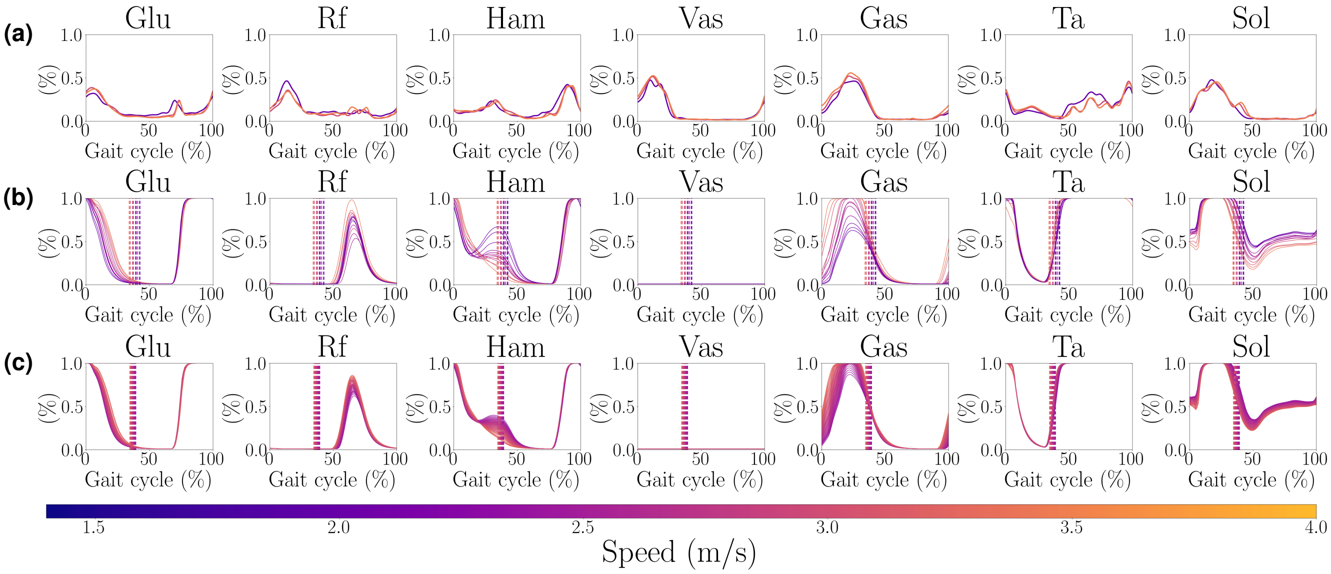

Figure S3. Muscular activations running Muscular activation at different speeds in comparison for a) Experimental data from Santuz et al.[2] at 2 m/s, 3 m/s and 3.5 m/s normalized for each trial to the maximum activation recorded for every individual muscle. For GLU, HAM, GAS, and VAS the mean activation of several muscles is shown (GLU: gluteus medius, gluteus maximus; HAM: semitendinosus, biceps femoris; VAS: vastus medialis, vastus lateralis; GAS: gastrocnemius medialis, gastrocnemius lateralis) b) Optimization with 71 parameters starting from Run Init $\mathbf{p}_r$ (0.45 m/s to 1.93 m/s) c) Modulation with 30 parameters (0.69 m/s to 1.44 m/s) starting from Run Init $\mathbf{p}_r$. Only muscles where Santuz et al.[2] provides experimental data are shown. Dotted vertical lines indicate toe-off.

Additional initial parameter sets

This section gives an overview of the results for the two additional initial parameter sets $\mathbf{p}_{w,alt}$ and $\mathbf{p}_{r,alt}$ that the pipeline was tested on. Fig. S4 displays the parameter values in comparison to the two main initial parameter sets, showing that the additional two parameter sets display large differences in parameter space with comparison to the main sets. Table S2 then lists the main results for optimization and modulation for all four initial parameter sets in comparison. For completeness Table S3 gives the variation explained by the first principal component PC1 for the PCA of each of the initial parameter sets. The results of PC1 were used to determine key parameters. As further results, the kinematics over speed for the additional walking (Fig. S5) and running (Fig. S6) parameter set, as well as the obtained stable velocities during modulation (Fig. S7) and the online modulation results (Fig. S8) are displayed. Finally, Fig. S9 and Fig. S10 provide some analysis of the differences of key reflexes between the different initial parameter sets.

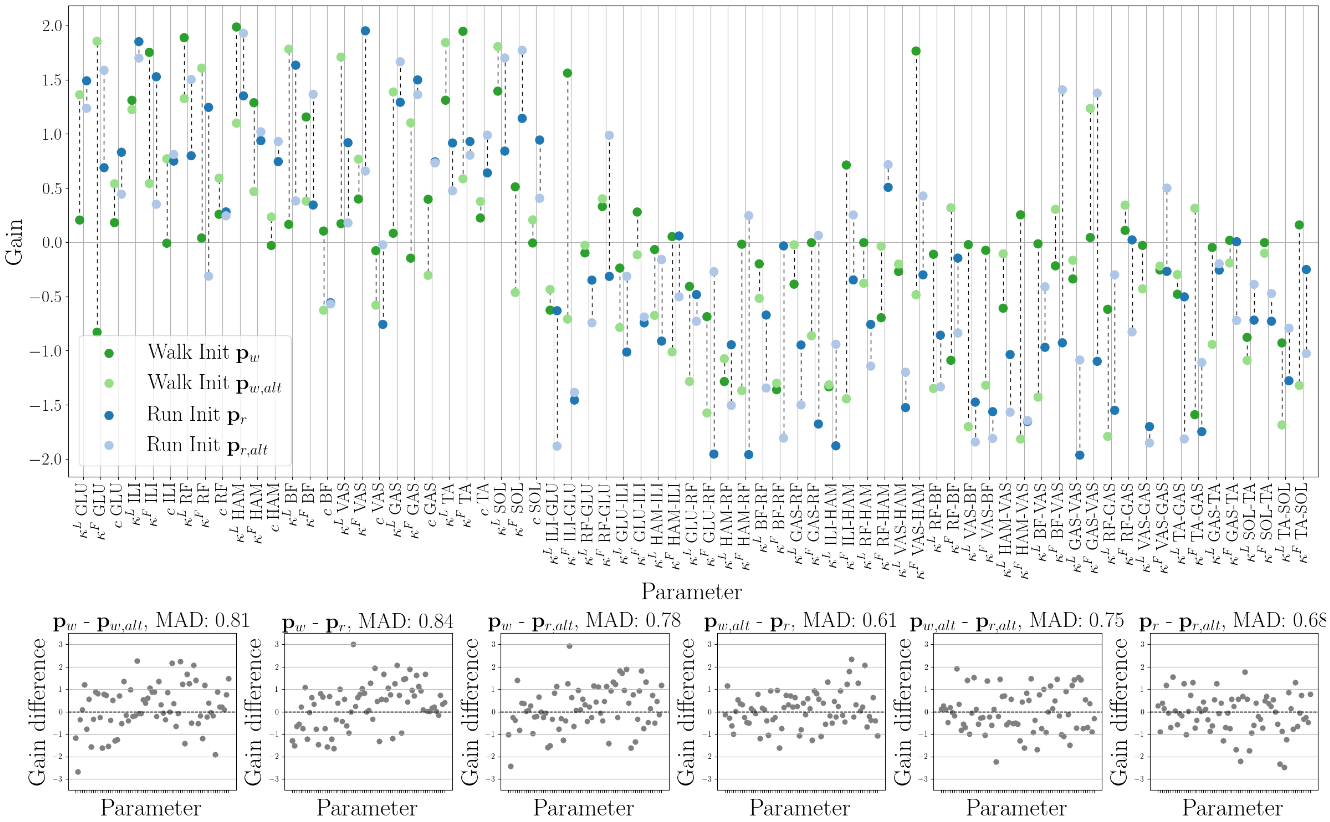

Figure S4. Comparison of initial parameter sets Parameter values of the used initial solutions for walking (green) and running (blue) (top) and pairwise comparison of all four initial parameter sets showing that they differ both within as well as between gait. The mean average difference (MAD) is given for each pair. The initial parameter sets have the following speeds: Walk Init $\mathbf{p}_w$: 1.10 m/s, Walk Init $\mathbf{p}_{w,alt}$: 1.22 m/s, Run Init $\mathbf{p}_r$ 2.48 m/s , Run Init $\mathbf{p}_{r,alt}$: 2.64 m/s.

Table S2. Overview speed ranges Obtained speed ranges for the four tested initial parameter sets: speed range and minimum/ maximum speed for optimization using all parameters (Opt 71), optimization using 30 parameters (Opt 30) and modulation using the identified 30 key parameters and a nonlinear regression of degree 3 (Mod 30). Gray cells are also shown in the main manuscript.

	**Walk Init $\mathbf{p}_w$**			**Run Init $\mathbf{p}_r$**		
	Opt 71	Opt 30	Mod 30	Opt 71	Opt 30	Mod 30
v_{range}(m/s)	1.48	1.25	0.75	1.40	1.36	0.76
v_{min}(m/s)	0.45	0.51	0.69	2.00	2.02	2.39
v_{max}(m/s)	1.93	1.76	1.44	3.40	3.38	3.16
	**Walk Init $\mathbf{p}_{w,alt}$**			**Run Init $\mathbf{p}_{r,alt}$**		
	Opt 71	Opt 30	Mod 30	Opt 71	Opt 30	Mod 30
v_{range}(m/s)	1.52	1.07	0.57	1.99	1.51	0.61
v_{min}(m/s)	0.45	0.60	0.83	1.41	1.80	2.23
v_{max}(m/s)	1.97	1.67	1.40	3.40	3.31	2.84

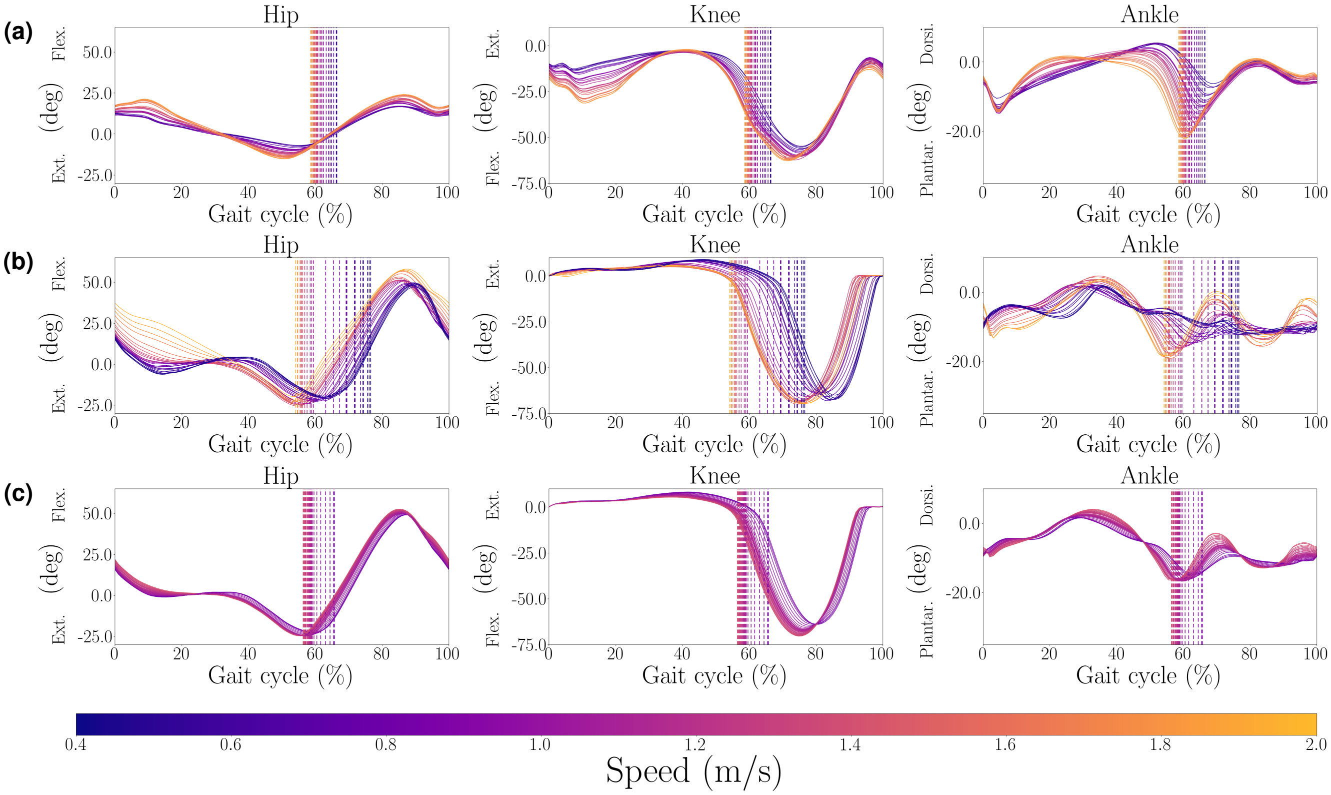

Figure S5. Walking kinematics Hip, knee, and ankle kinematics at varying walking speeds: a) Experimental data from Camargo et al.[1] (0.5 m/s to 1.85 m/s) b) Walk Init $\mathbf{p}_{w,alt}$: Optimization with 71 parameters (0.45 m/s to 1.97 m/s) c) Walk Init $\mathbf{p}_{w,alt}$: Modulation with 30 parameters (0.83 m/s to 1.40 m/s). Dotted vertical lines indicate toe-off. The zero configuration represents standing position.

	Walk Init $\mathbf{p}_w$	Run Init $\mathbf{p}_r$	Walk Init $\mathbf{p}_{w,alt}$	Run Init $\mathbf{p}_{r,alt}$
PC1	0.47	0.45	0.4	0.54

Table S3. Results principal component analysis (PCA) Explained variance for the first principal component (PC1) for each of the four different initial parameter sets.

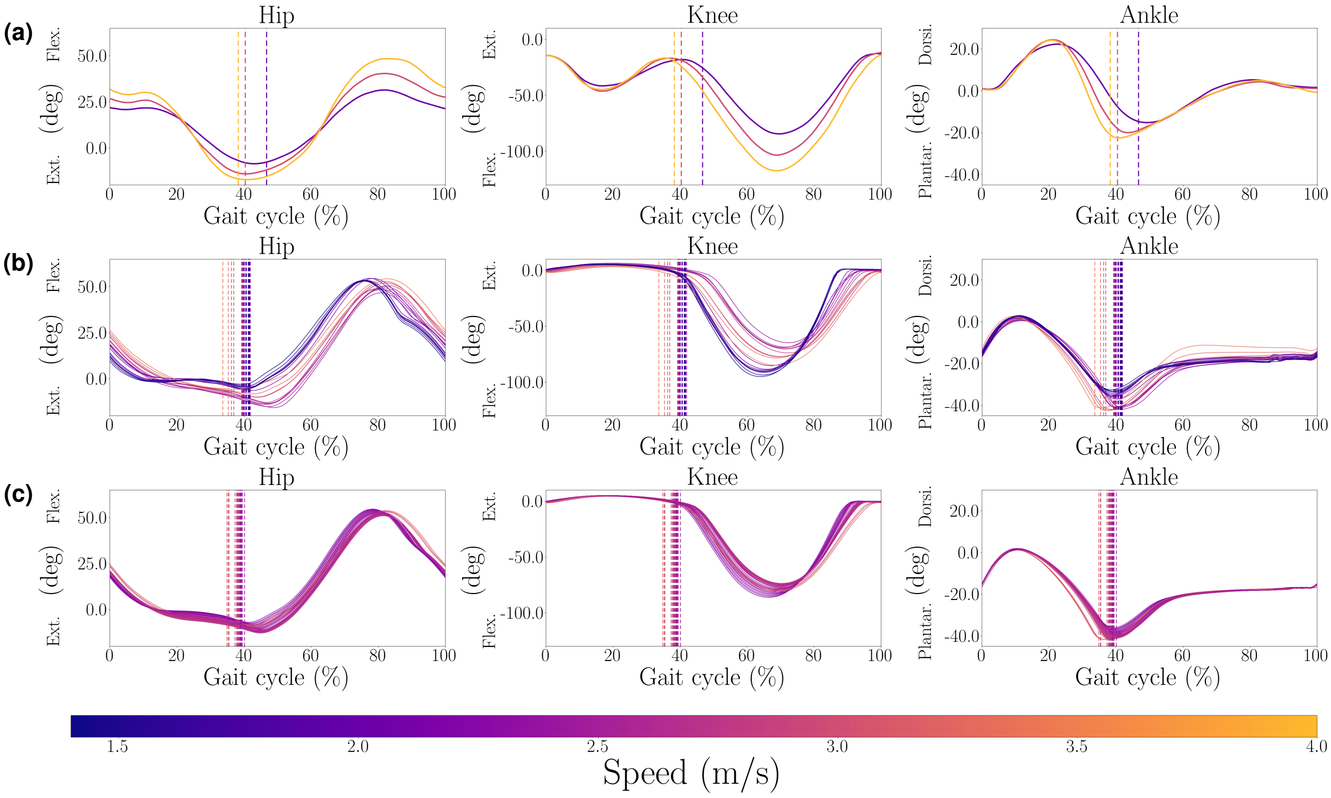

Figure S6. Running kinematics Hip, knee, and ankle kinematics at varying running speeds a) Experimental data of running at 2 m/s, 3 m/s and 4 m/s from Hamner et al.[3] b) Run Init $\mathbf{p}_{r,alt}$: Optimization with 71 parameters (1.41 m/s to 3.40 m/s) and c) Run Init $\mathbf{p}_{r,alt}$: Modulation with 30 parameters (2.23 m/s to 2.84 m/s). Dotted vertical lines indicate toe-off. The zero configuration represents standing position.

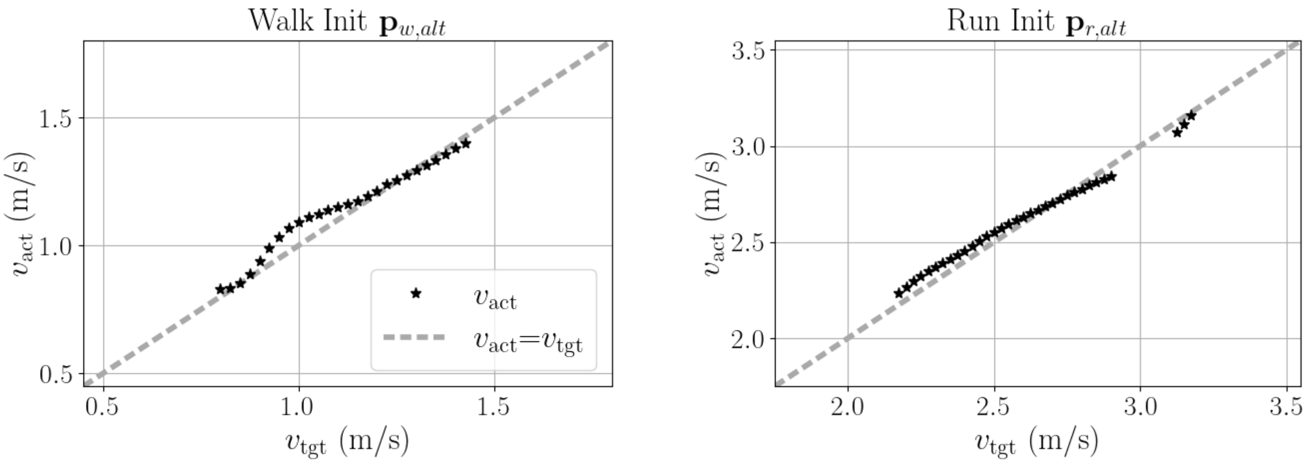

Figure S7. Offline modulation Actual velocity v_{act} versus target velocity v_{tgt}(black) for the modulation strategy with 30 parameters and deg 3 for the two additional initial parameter sets ($\mathbf{p}_{w,alt}$ and $\mathbf{p}_{r,alt}$). v_{act} is only calculated for stable solutions with $d_{\text{sim}} = 50\,\text{s}$.

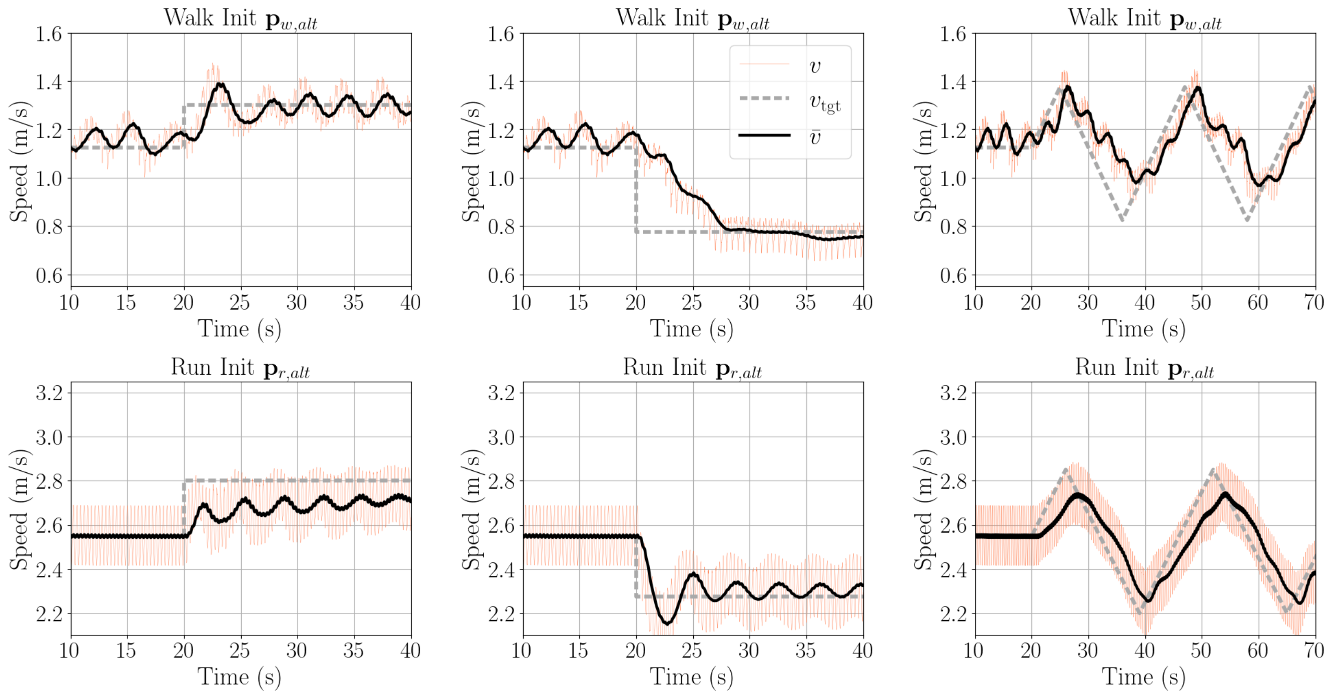

Figure S8. **Online modulation** Prescribed target velocity v_{tgt} (grey), torso velocity v (orange) and average velocity $\bar{v}$ of the torso (calculated with a moving average of 1 s, black) for the additional initial parameter sets for walking (top row) and running (bottom row) for maximal (left) and minimal (center) steps as well as ramps (right) within the stable modulation speed range.

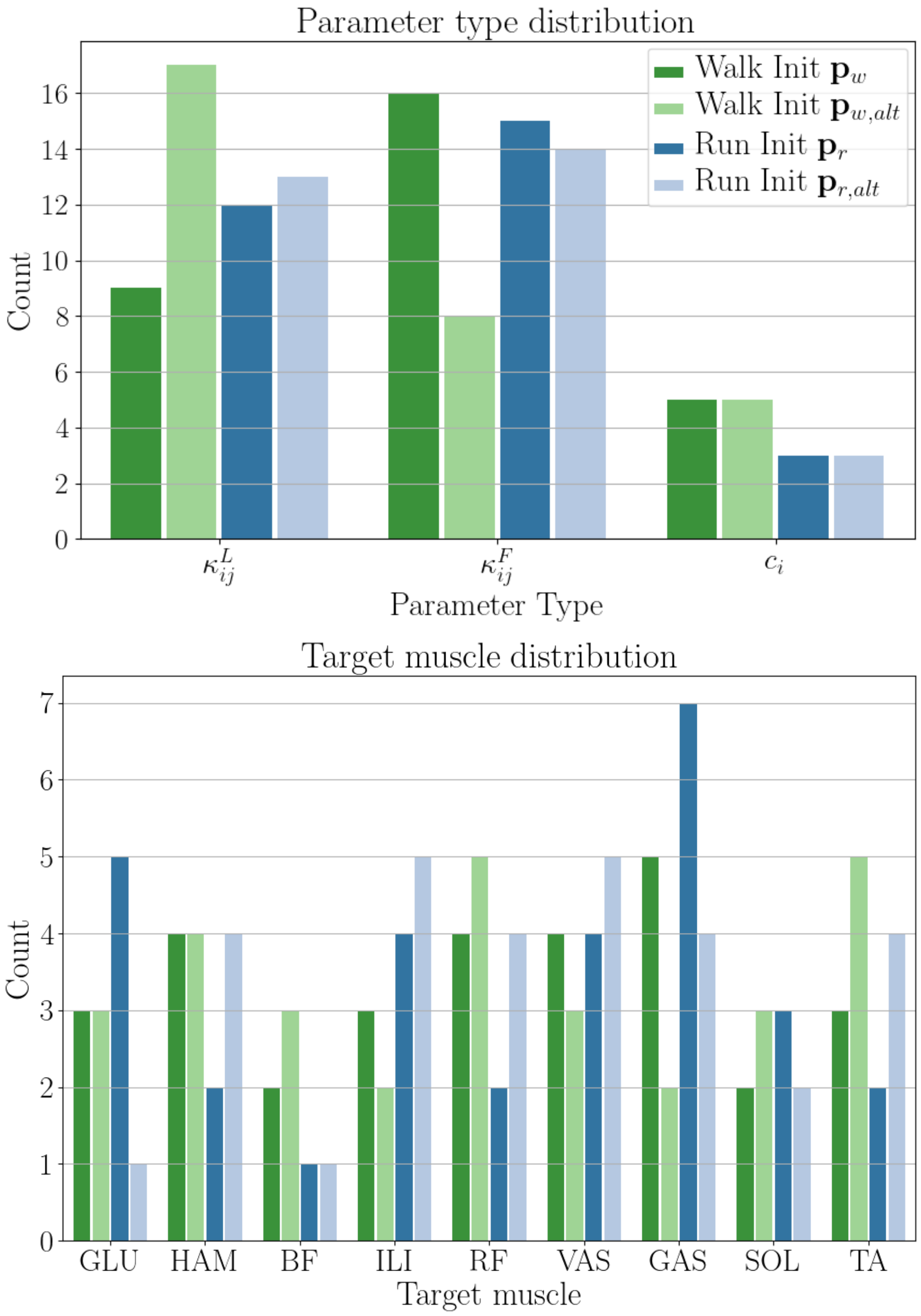

Figure S9. Analysis key parameters The found 30 key parameters per initial parameter set are analysed by parameter type (length κ_{ij}^L, force κ_{ij}^F or constant offset c_i, top) and target muscle (bottom). For each initial parameter set, all muscles receive at least the input of one key reflex and length, force as well as offsets are present in all sets.

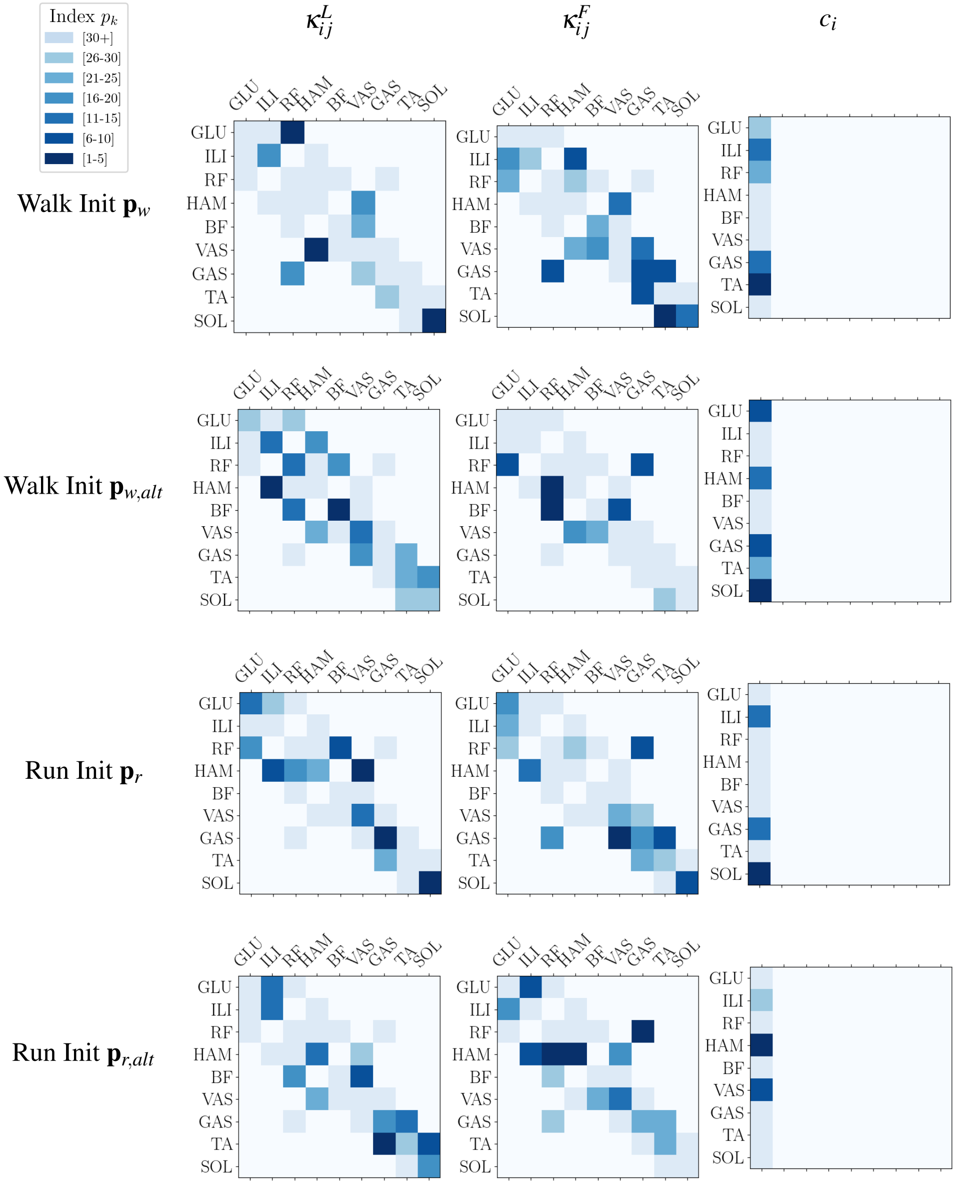

Figure S10. Key parameters Comparison of the found key parameters for starting from the four initial parameter sets (from top to bottom), split in length κ^L_{ij} (left) and force κ^F_{ij} (center) reflexes as well as offsets c_i (right). Colors indicate the index of the key parameters p_k (the darker the earlier it is included, i.e., when only 5 key parameters are chosen). Light-blue indicates that the reflex was not included in the 30 parameters.

Experimental setting for transition

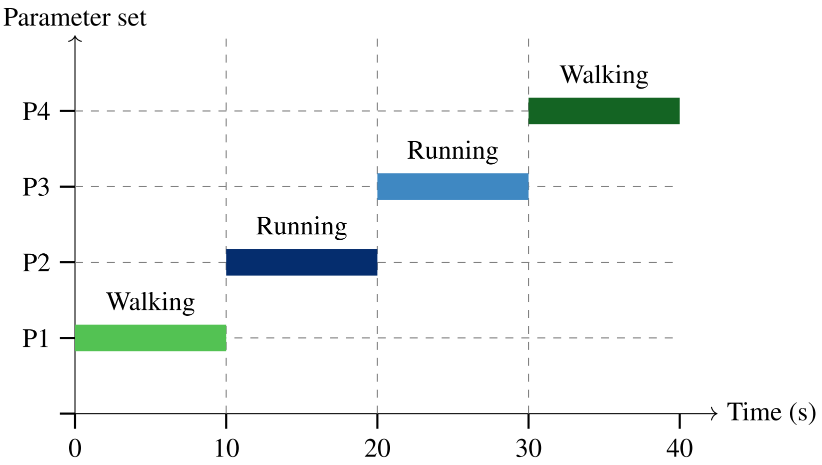

Figure S11. Gait transition experiments Approach employed for experiments on gait transitions between walking and running. Four different parameter sets of 71 parameters each are optimized together, yielding a total of 284 optimization parameters. The controller abruptly switches between the four sets at fixed intervals of 10 s. The starting points of the iterative optimization process are manually-picked found solutions of fast walking and slow running and the parameters are then optimized to achieve stable gait throughout all transitions. At the end this results in a sequence of walk-run-run-walk.

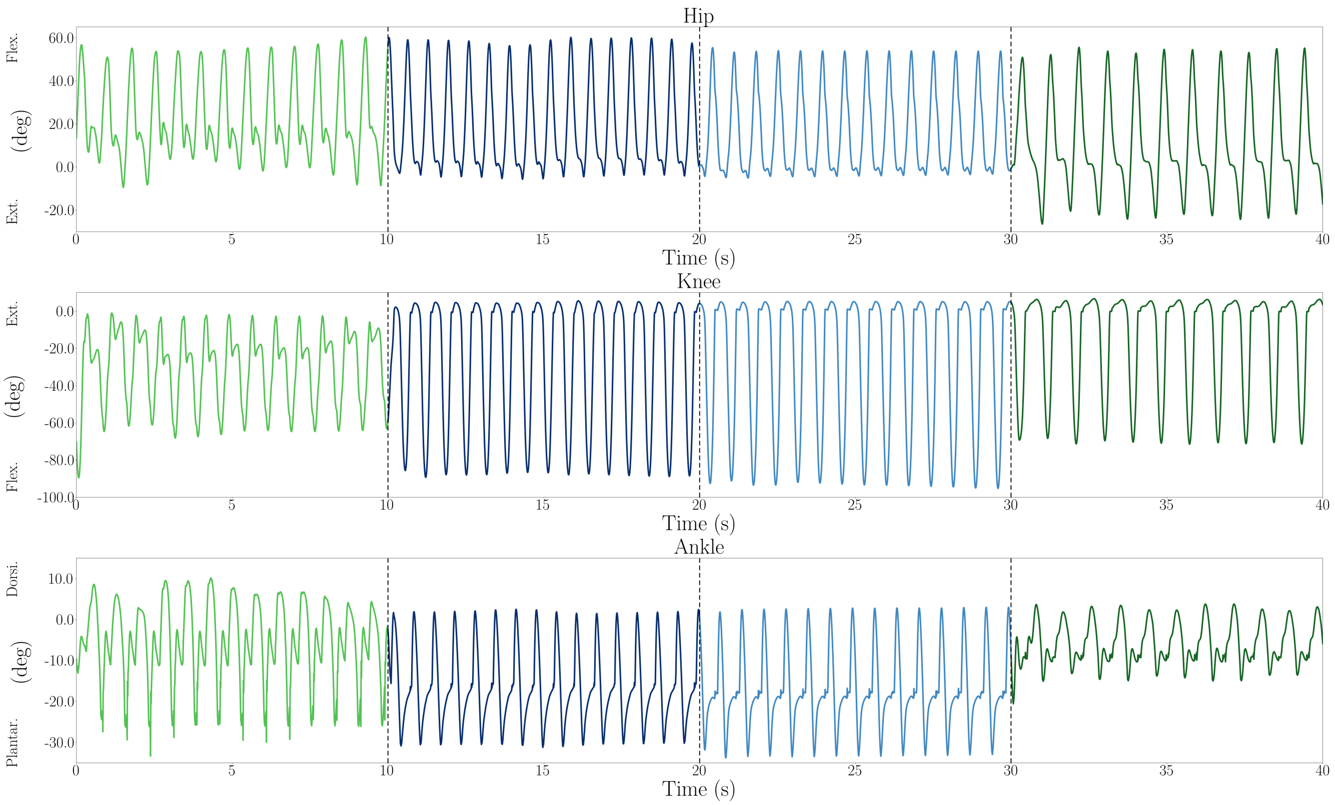

Figure S12. Joint trajectories gait transition experiments Joint angles of hip (top), knee (middle) and ankle (bottom) joints for the tested walk-run-run-walk transition (compare Fig. S11). Dashed vertical lines indicate the time where the controller parameters are changed.

References

1. Camargo, J., Ramanathan, A., Flanagan, W. & Young, A. A comprehensive, open-source dataset of lower limb biomechanics in multiple conditions of stairs, ramps, and level-ground ambulation and transitions. *J. Biomech.* **119**, 110320 (2021).

2. Santuz, A. *et al.* Lower complexity of motor primitives ensures robust control of high-speed human locomotion, DOI: 10.5281/zenodo.6655814 (2022).

3. Hamner, S. R. & Delp, S. L. Muscle contributions to fore-aft and vertical body mass center accelerations over a range of running speeds. *J. biomechanics* **46**, 780–787 (2013).
